# Neuropsychological Learning Deficits as Predictors of Treatment Outcome in Patients with Eating Disorders

**DOI:** 10.3390/nu13072145

**Published:** 2021-06-23

**Authors:** Ignacio Lucas, Romina Miranda-Olivos, Giulia Testa, Roser Granero, Isabel Sánchez, Jessica Sánchez-González, Susana Jiménez-Murcia, Fernando Fernández-Aranda

**Affiliations:** 1 Department of Psychiatry, Bellvitge University Hospital-IDIBELL, 08907 L’Hospitalet de Llobregat, Spain; ilucas@idibell.cat (I.L.); rmiranda@idibell.cat (R.M.-O.); gtesta@idibell.cat (G.T.); roser.granero@uab.cat (R.G.); isasanchez@bellvitgehospital.cat (I.S.); jsanchezg@bellvitgehospital.cat (J.S.-G.); sjimenez@bellvitgehospital.cat (S.J.-M.); 2 Psychiatry and Mental Health Group, Neuroscience Program, Institut d’Investigació Biomèdica de Bellvitge-IDIBELL, 08907 L’Hospitalet de Llobregat, Spain; 3 Ciber Fisiopatología Obesidad y Nutrición (CIBERObn), Instituto de Salud Carlos III, 08907 L’Hospitalet de Llobregat, Spain; 4 Department of Psychobiology and Methodology of Health Sciences, Autonomous University of Barcelona, 08193 Barcelona, Spain; 5 Department of Clinical Sciences, School of Medicine, University of Barcelona, 08907 L’Hospitalet de Llobregat, Spain

**Keywords:** eating disorders, decision making, learning, treatment outcome

## Abstract

Eating disorders (EDs) are severe psychiatric illnesses that require individualized treatments. Decision-making deficits have been associated with EDs. Decision-making learning deficits denote a lack of strategies to elaborate better decisions that can have an impact on recovery and response to treatment. This study used the Iowa Gambling Task (IGT) to investigate learning differences related to treatment outcome in EDs, comparing between patients with a good and bad treatment outcome and healthy controls. Likewise, the predictive role of impaired learning performance on therapy outcome was explored. Four hundred twenty-four participants (233 ED patients and 191 healthy controls) participated in this study. Decision making was assessed using the Iowa Gambling Task before any psychological treatment. All patients received psychological therapy, and treatment outcome was evaluated at discharge. Patients with bad outcome did not show progression in the decision-making task as opposed to those with good outcome and the healthy control sample. Additionally, learning performance in the decision-making task was predictive of their future outcome. The severity of learning deficits in decision making may serve as a predictor of the treatment. These results may provide a starting point of how decision-making learning deficits are operating as dispositional and motivational factors on responsiveness to treatment in EDs.

## 1. Introduction

Eating disorders (EDs) are important psychiatric illnesses that involve abnormal eating behavior. Patients affected with EDs may present excessive concern over food, body weight, and shape dissatisfaction. These conditions could also lead to serious physical problems and impaired psychosocial functioning [1]. Moreover, there is an increased risk of suicide in people with EDs compared to the non-ED population [2,3,4,5]. A recent systematic review regarding the diagnosis prevalence of EDs established that worldwide, around 8.4% of women and 2.2% of men will be diagnosed with this condition at some point in their lifetime [6]. The main treatments for EDs, which are based on cognitive–behavioral therapy (CBT), have been demonstrated to be useful in reducing symptoms [7,8]; however, these current treatments have not always reported successful outcomes [9,10,11,12].

A systematic review [13] reported ED remission rates between 18% and 62%. Several individual circumstances might underlie the response to treatment in EDs, increasing the risk of having bad treatment outcomes, resulting in low remission rates or poor adherence to treatment [14,15,16,17,18,19]; therefore, assessing which functions act as predictors for the treatment outcome of the ED is crucial in order to design optimized individual treatments [20,21,22].

Some of the most studied cognitive features in patients with EDs are their executive function impairments in comparison to the healthy population [16,23,24,25,26,27,28]. Executive functions optimize cognitive processes to solve demanding situations where instinct or intuition is insufficient [29]. Complex cognitive processes, such as decision making, are strongly related to executive functions [30]. Decision making involves high-level processes, including option generation, evaluation of risks and consequences, and choosing between different possibilities in order to achieve a certain personal objective [31]. Therefore, decision-making processes require complex high-level processing to make advantageous decisions taking different variables into account. These processes are commonly related to prefrontal cortex activity [32,33]. Psychiatric illnesses, such as EDs, are usually associated with significant impairments in prefrontal, fronto-limbic, and fronto-striatal neural systems [34].

Even though each ED subtype has been related to its own specific neurocognitive impairments [35], decision-making deficits have been found among all ED conditions [25,27,36,37,38,39,40]. Patients with EDs reported poor learning during decision-making paradigms [41,42], showing a tendency to persist in decisions/choices, despite negative consequences. Learning deficits in the decision-making tasks of patients diagnosed with EDs may be related to an excessive sensitivity to reward or punishment, which could be associated with the persistence of their dysfunctional behavior [42]. Some studies have hypothesized that in EDs, as reported in obsessive-compulsive disorders, observed impairments in decision making may be related to biological markers [26,43]; however, decision-making deficits in EDs do not have to be considered a completely permanent feature. Neurocognitive training on executive functions has been tested in patients with EDs, showing improvement in cognitive flexibility, inhibitory control, and working memory [16]. Furthermore, in another study, patients with anorexia nervosa showed great improvement in decision making after CBT treatment in patients in full remission of their ED symptoms but not in patients with no remission [40]. Just as patients with EDs who improve their symptoms showed an improvement in their performance post-treatment, it could be expected that better decision making at baseline would also predict a better treatment outcome; however, the literature examining neurocognitive predictors of treatment outcome in EDs is scarce [44] and there is a lack of studies focusing on neuropsychological profiles as predictors of therapy outcome [45]. Cavedini et al. [14] observed how the function of decision making might be linked to treatment outcomes in women with anorexia nervosa. Still, they pointed toward the necessity of understanding which neurocognitive feature linked to decision making can be used as a criterion for selecting the proper treatment.

Based on the facts described above, this research was designed with two aims: first, to assess baseline learning differences related to decision-making between patients with EDs who recovered from their symptoms and those who did not; second, to explore the predictive capacity of impaired learning performance on therapy outcome.

According to the above-mentioned aims, we propose two hypotheses. First, if learning decision-making skills influence treatment efficacy, EDs with bad treatment outcomes will show impaired learning performance, even before the treatment. Second, if there is an impaired neurocognitive functioning in ED patients with bad treatment results, the decision-making learning skill will help discriminate between having good or bad treatment outcomes.

## 2. Materials and Methods

### 2.1. Participants

A total of 424 participants were included in the present study: 341 women and 83 men, with a ratio similar to recent studies [6]. The ED group contained 190 women and 43 men, with a mean age of 30.52 (SD = 10.9), whereas the healthy control (HC) group (151 women and 40 men) had a mean age of 25.65 (SD = 8.5). In terms of the highest level of education, for the HC group, 5.8% attained a primary education, 56% attained a secondary education, and 38.2% attained a tertiary degree. For the ED group, 35.2% attained a primary education, 40.8% attained a secondary education, and 24% attained a tertiary degree. Table S1 (Supplementary Material) contains the sociodemographic characteristics of the groups at baseline. To avoid potential biases in the results, all the comparisons were adjusted for the covariates of age and education level at baseline. Patients with EDs were recruited from the Eating Disorders Unit at Bellvitge University Hospital in Barcelona, Spain. All patients within the ED group met the Diagnostic and Statistical Manual of Mental Disorders (DSM-5; American Psychiatric Association, Philadelphia, PA, USA) [46] criteria for EDs, following standardized structured interviews. The ED group was composed of 85 patients with anorexia nervosa (AN) restrictive subtype, 41 patients with AN bulimic/purging subtype, 44 with bulimia nervosa (BN), 45 patients with binge eating disorder (BED), and 18 patients with other specified feeding or eating disorder (OSFED). Once diagnosed, they were asked to voluntarily participate in this study. Neuropsychological and clinical assessments were conducted in the first week of their treatment. The exclusion criteria for the HC group were a body mass index below 18.5 or above 25 and a lifetime history of EDs, according to a semi-structured interview and following DSM-5 diagnostic criteria.

Data were collected between May 2008 and November 2020. All participants were adults, received information about the procedure, and signed an informed consent form. All procedures were approved by the Ethical Committee of the Bellvitge University Hospital in accordance with the Helsinki Declaration of 1975 as revised in 1983.

### 2.2. Procedure

Participants completed a computerized version of the Iowa Gambling Task (IGT) [47]. Additionally, the patients’ psychopathology symptoms were evaluated via the Spanish version of the Symptom Checklist-Revised (SCL-90-R) [48], and their ED symptoms were assessed with the Spanish version of the Eating Disorders Inventory-2 (EDI-2) [49]. All these evaluations were conducted prior to the psychological treatment.

#### 2.2.1. Decision-Making Assessment

The computerized version of the IGT was used to assess decision-making processes [50]. This task consists of 100 trials in which the participants must draw a card from one of the four presented decks (A, B, C, and D). Each card represents a monetary gain but can also result in monetary loss. There are two advantageous decks and two disadvantageous ones. The first ones produce less monetary incomes but with an overall gain, whereas the second presents larger gain amounts and an overall monetary loss. The participant has to gain as much as possible by the end of the task. It is subdivided into five blocks of twenty trials performed consecutively. The first blocks allow measuring decision making under ambiguity, whereas in the last blocks, the task switches to decision making under risk because the rules may have been figured out [51].

The test score for each block (IGT-1, 2, 3, 4, and 5) is calculated by subtracting the number of choices from disadvantageous decks to the number of choices from advantageous decks draws. The total task score (IGT-Total) is calculated by adding the scores of the five blocks. The task also allows us to calculate a learning score (IGT-Learning) and a risk score (IGT-Risk) [42]. IGT-Learning is calculated with the difference between the scores of the two first blocks and the two last ones. This approach/procedure allows us to assess the differences between the first and final blocks. The first blocks are assessed because the participant has not learned which decks are advantageous and disadvantageous; the last blocks are assessed because the experience gained through the trial can produce changes in choice patterns. Furthermore, IGT-Risk considers only the scores from the two last blocks, where a participant could have already detected which decks involve a risky choice.

#### 2.2.2. Treatment

As described elsewhere [9,52], patients diagnosed with AN attended a day hospital treatment program, including CBT group therapy sessions, lasting 90 min each, for 15 weeks. Treatment for the other ED diagnosis (BN, BED, and OSFED) consisted of 16 weekly outpatients CBT group therapy sessions, lasting 90 min each. Patients were re-evaluated at discharge and categorized as either in full remission (i.e., total absence of symptoms meeting criteria for at least 4 weeks), partial remission (i.e., a substantial symptomatic improvement but with residual symptoms), and non-remission. These categories were previously used as the threshold to assess treatment outcomes in patients with EDs [9,19,52]. The treatment outcomes categories were based on the judgments of senior clinical staff considering normalization of nutritional dietary patterns, frequency of binge episodes and compensatory behaviors, weight restoration, and improvement in attitudes regarding weight and shape. Voluntary treatment discontinuation was categorized as dropout (i.e., not attending treatment for at least three consecutive sessions). Patients were subdivided into two groups depending on their treatment outcome. Those who showed full or partial remission of their symptoms were included in the good outcome group (*n* = 166; 71.2%), and those who did not show remission or abandoned the treatment were included in the bad outcome group (*n* = 67; 28.8%). The treatment results obtained were similar to those reported previously [53].

### 2.3. Data Analysis

Statistical analysis was done with Stata16 for windows (College Station, TX, USA). The association between the baseline measures with the CBT efficiency (bad versus good outcome) was based on the chi-square test (χ^2^) for categorical measures and analysis of variance (ANOVA) for quantitative measures. An increase in the Type-I error due to the multiple significance tests was based on the Finner method [54], which is a family-wise procedure that has proved more powerful than the standard Bonferroni correction.

The comparison of the learning curves in the IGT was based on 3 × 5 mixed ANOVA (adjusted by the participants’ age and education level), which is defined as the between-subjects factor of the group (bad CBT outcome, good CBT outcome, and control condition) and as the within-subjects factor for the score in each block. Polynomial contrasts for the within-subject factor assessed linear, quadratic, cubic, and quartic trends in the learning curves. Comparing the IGT-Learning score between the three groups was also based on analysis of variance, which was adjusted by age and education (ANCOVA).

The discriminative capacity of the IGT-Learning score to discriminate between good versus bad outcomes in the CBT was based on Receiver Operating Characteristics (ROC) analysis. This methodology is used in clinical areas to obtain the optimal cut-off in measurement tools using an external reference criterion. In this work, ROC analysis was applied within the ED subsample to obtain the best cut-off in the IGT index to discriminate between patients with bad versus good CBT outcomes. Since selecting the optimal cut-off depends on the prevalence of the criteria and the costs/risks of false classifications [55], the analysis was performed considering a distribution for the CBT outcome equal to the sample and a cost for a false negative double compared to the cost for a false positive.

Logistic regression valued the capacity of the optimal cut-off point in the IGT-Learning global measure to differentiate between bad and good outcomes. Goodness-of-fit was assessed with the Hosmer and Lemeshow test.

In this study, the effect size was based on the eta-squared coefficient (η^2^) for quantitative measures (values of 0.06, 0.10, and 0.25 were interpreted as low–poor, moderate–medium, and large–high effect size) [56], and in Cramer’s-V coefficient for categorical (values of 0.10, 0.30, and 0.50 were interpreted as low–poor, moderate–medium, and large–high effect size) [57].

## 3. Results

### 3.1. Comparison of the IGT Measures between the Groups

**Figure 1:**
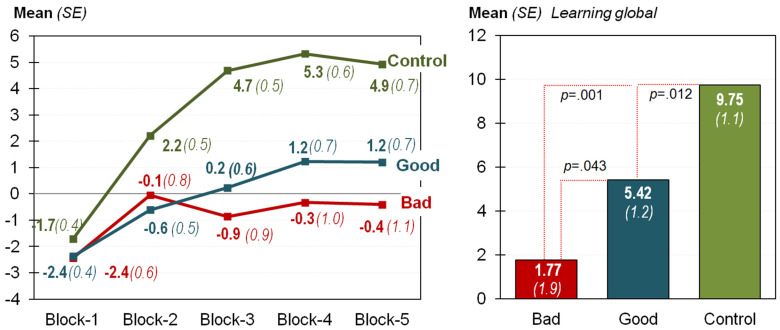
Iowa Gambling Task (IGT) performance–learning curves (**left**) and IGT global scores by group (**right**). Note. Sample size *n* = 424. SE: standard error.

Table 1 contains the results obtained in the mixed ANOVA (adjusted by age and education) comparing the proficiency in the IGT between the groups (see also the first panel in Figure 1). The interaction of the within- and between-subjects factors was statistically significant (*F* = 4.09, *p* < 0.001, η^2^ = 0.019), indicating that the learning curves had a specific shape depending on the group. No statistically significant differences between the blocks were found among patients in the bad outcome group (*F* = 1.63, *p* = 0.166, η^2^ = 0.015), suggesting the absence of a learning curve. Within patients with a good outcome, significant linear (*F* = 23.3, *p* < 0.001, η^2^ = 0.124) and quadratic (*F* = 6.49, *p* = 0.012, η^2^ = 0.038) trends appeared: increasing means with blocks were registered (from −2.4 in block 1 to 1.2 in block 5), the difference being lower comparing blocks 4 versus 5 (1.23 versus 1.21). The same pattern was obtained in the control group: significant linear (*F* = 79.71, *p* < 0.001, η^2^ = 0.296) and quadratic (*F* = 27.99, *p* < 0.001, η^2^ = 0.128) trends.

Table 2 contains the results of the ANCOVA (adjusted by age and education) comparing the IGT-Learning score between the groups (see the second panel in Figure 1). Statistical differences between the groups appeared (*F* = 7.14, *p* < 0.001, η^2^ = 0.124). Pairwise comparisons (contrasts between the groups) also achieved differences between all the groups.

### 3.2. Discriminative Capacity of the IGT-Learning Score

**Figure 2:**
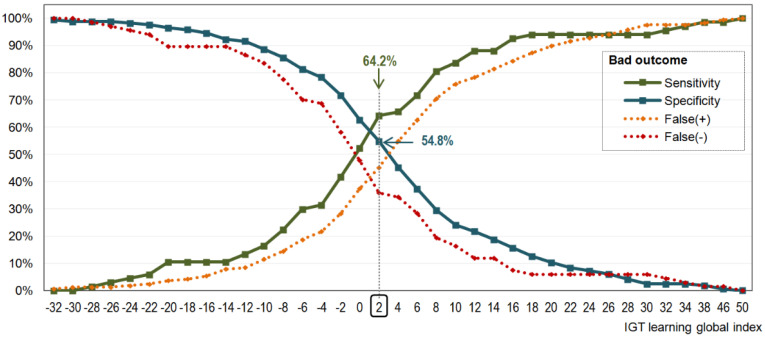
Valuation of the IGT-Learning raw score to predict the treatment outcome. Note. Results obtained for the ED subsample (*n* = 233).

Figure 2 contains the results of the ROC analysis obtained in the ED subsample. The optimal cut-off point in the IGT-Learning index to discriminate between good and bad CBT outcomes was 2, which achieved a sensitivity (Se), or true positive rate, of 64.2% and a specificity (SP), or true negative rate, equal to 54.8%.

**Figure 3:**
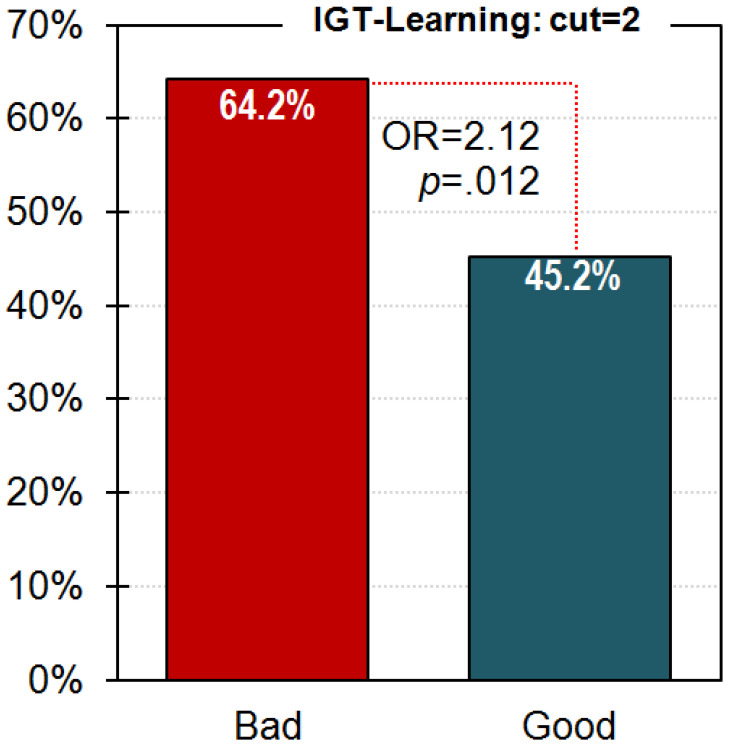
Capacity of the IGT-Learning score to predict the treatment outcome. Each bar represents the percentage of participants with poor IGT-Learning in each group with a cut-off point equal to 2. Note. Results obtained for the ED subsample (*n* = 233).

Figure 3 shows the percentage of patients with a poor performance in the IGT in each group (based on the classification obtained for the cut-off = 2 in the global learning measure). The logistic regression (adjusted by age and education) valuing this cut-off’s capacity for differentiating between the two groups achieved a significant parameter for differentiating between bad versus good groups (B = 0.754, SE = 0.301, OR = 2.12, *p* = 0.012). Goodness-of-fit was achieved (Hosmer and Lemeshow test: χ^2^ = 5.95, df = 8, *p* = 0.653).

### 3.3. Variables Associated with the CBT Outcome

Table 3 contains the comparison between patients classified according to the CBT outcome (bad versus good) at baseline. No differences were found between groups in any of the variables.

## 4. Discussion

We examined baseline differences in decision making in patients with EDs, differentiating between those who improved vs. those who did not after CBT, and analyzed its therapy outcome predicting value. As the first objective, our study addressed whether ED patients with different outcomes present learning differences related to decision making before the treatment. This study’s main results showed how both the patients with good outcomes and the healthy control group showed a learning curve through the IGT task; however, the bad outcome group was the only group that did not show progression across the blocks. Based on these results, the first hypothesis is verified, as different outcomes present differences in learning, even before the intervention. The second main finding was that the IGT-Learning score predicted treatment outcome. These findings support our second hypothesis, as the capacity of learning through a decision-making task seems to discriminate between having a successful or a bad treatment outcome. There would be a chance that these learning deficits were related to higher depressive symptoms; nevertheless, there were no observed differences in depression between ED groups.

These results fit not only with previous studies that point toward decision-making deficits in patients with EDs [25,27,36,38,39,40] but also with the ones that report how individual differences correlate with distinct treatment outcomes [9,15,16,17,18,19]. Regarding a previous study that presented decision making as a predictor of treatment outcome in EDs [14], our study reported its predictive value using a bigger sample, with patients of both sexes and with different EDs subtypes. In addition, among the neuropsychological variables that discriminate between the treatment results, the learning skills showed differences depending on therapy outcomes and are good predictors of the treatment result. It is noteworthy to mention that patients with EDs who had a poor treatment outcome did not show changes in their answers across the IGT blocks; this could mean that perhaps they neither changed their behavior due to immediate rewards (as in disadvantageous decks) nor to delayed recompenses (as in advantageous decks) [36]. According to Hiroto and Seligman [58], this lack of change is probably related to learned helplessness, and therefore, they may not feel capable of changing the result of the task through their decisions. This behavior could explain why they do not believe in the possibility of improving their symptoms with psychological intervention, leading to poor treatment efficacy and less treatment adherence. Steward et al. [40] reported how patients with EDs who recover from their symptoms also improve their performance in decision-making tasks; therefore, they enhance their learning skills. If that is true, a potential treatment effect would be a patient believing in their ability to change negative situations via their actions and decisions. There were no observed differences in ED symptoms nor in general psychopathology, so, in this sample, the different treatment outcomes do not seem to be directly related to these parameters.

Previous research showed how patients with EDs tend to report high levels of sensitivity to punishment [42,59,60]; however, in our study, some of them still did not seem to learn from the negative feedback; this may be due to the fact that despite stimuli producing a great emotional impact, those patients do not change their behavior because they do not believe they can change situations via their decisions. The main characteristic of learned helplessness is that it highly correlates with depressive states [61,62]. Nevertheless, regarding our results, these learning impairments would be related to a worse treatment outcome independently from the depressive symptoms. The patients with EDs who show impaired learning behaviors and tend to have negative treatment outcomes would need to change their belief in the possibility of improving their symptoms; therefore, individualized treatments for those patients will require focusing on improving their locus of control.

Our study has certain limitations, and the results and conclusions of our study must take these into account. First, using a neuropsychological task such as the IGT may not be practical for the clinical assessment; it would be necessary to design more accessible tools to assess these impairments. Second, our sample size was limited to test the predictive role of IGT performance across ED subtypes. Therefore, inferences emerging from these results must be interpreted with caution considering no discrimination by ED diagnosis. Future studies with larger samples could elucidate the predictive role of decision-making learning in each ED subtype. Third, as seen in other psychological disorders, impaired motivation may influence the performance in cognitive tasks [63]. Future research should include some motivational measure to assess this effect. Fourth, it will still be necessary to evaluate whether there are differences between those patients who do not recover from their symptoms and those that show poor treatment adherence. This study presents an understanding of how neurocognitive deficits may underlie possible treatment outcomes in ED. Future studies should consider our results to develop individualized treatments so that patients with different features and symptoms can benefit from the treatment.

## 5. Conclusions

In sum, our results show how ED treatment outcomes could be related to cognitive functioning even before the treatment, as patients with different outcomes seem to present different learning skills related to decision making. This learning skill also demonstrated a predictive value for the treatment outcome, possibly indicating that patients who do not change their behavior despite its consequences tend to present greater difficulties with the treatment. It may indicate that these patients show a lack of belief in changing their situation through their behavior. These results point toward the importance of taking into account neuropsychological variables to develop and apply individualized treatments that successfully deal with EDs.

## Figures and Tables

**Table 1:** Performance learning curves in the Iowa Gambling Task (2 × 5 ANOVA adjusted by age and education).

	IGT Raw Scores											
	Block 1			Block 2			Block 3		Block 4		Block 5	
Group (outcome)	Mean	SD		Mean		SD	Mean	SD	Mean	SD	Mean	SD
Bad (*n* = 67)	−2.45	3.85		−0.05		4.28	−0.87	5.82	−0.32	7.78	−0.41	7.64
Good (*n* = 166)	−2.38	4.66		−0.61		5.53	0.24	5.87	1.23	7.42	1.21	8.45
Control (*n* = 191)	−1.72	5.92		2.22		7.06	4.68	8.35	5.32	9.03	4.93	9.90
Multivariate tests	*F*	*df*		*p*		η^2^						
Int. BxG	4.09	8; 419		0.001 *		0.019						
Block	0.80	4; 419		0.401		0.002						
Group	22.34	2; 419		0.001 *		0.096						
Factor Block Within Group	*F*	*p*		η^2^								
Bad	1.63	0.166		0.015								
Good	6.51	0.001 *		0.059								
Control	30.89	0.001 *		0.229								
Polynomial contrast for Block	Linear (order 1)			Quadratic (order 2)			Cubic (order 3)			Quartic (order 4)		
	*F*	*p*	η^2^	*F*	*p*	η^2^	*F*	*p*	η^2^	*F*	*p*	η^2^
Group: bad	1.14	0.289	0.017	3.94	0.051	0.056	1.20	0.277	0.018	1.73	0.193	0.026
Group: good	23.32	0.001 *	0.124	6.49	0.012 *	0.038	0.15	0.701	0.001	1.02	0.314	0.006
Group: control	79.71	0.001 *	0.296	27.99	0.001 *	0.128	0.55	0.457	0.003	0.53	0.468	0.003

Note. SD: standard deviation; * Bold: significant comparison (0.05 level); η^2^: partial eta-squared.

**Table 2:** Comparison of the IGT learning global score: ANCOVA adjusted by age and education.

	Bad Outcome		Good Outcome		Control	
Descriptives	Mean	SD	Mean	SD	Mean	SD
	1.77	14.04	5.42	13.15	9.75	16.67
Factor group	*F*	*df*	*p*	η^2^		
	7.14	2; 423	0.001 *	0.033		
Pairwise comparisons	*F*	*p*	η^2^			
Bad vs. good	4.84	0.043 *	0.014			
Bad vs. control	12.93	0.001 *	0.030			
Good vs. control	6.44	0.012 *	0.015			

Note. SD: standard deviation; * Bold: significant comparison (0.05 level); η^2^: partial eta-squared.

**Table 3:** Association between baseline measures with the cognitive–behavioral therapy outcome.

		Bad Outcome (*n* = 67)		Good Outcome (*n* = 166)			
Sex		*n*	%	*n*	%	*p*	V
	Women	59	88.1%	131	78.9%	0.103	0.202
	Men	8	11.9%	35	21.1%		
		Mean	SD	Mean	SD	*p*	η^2^
Chronological age (years-old)		28.99	9.50	31.13	11.39	0.174	0.008
Duration of disorder (years)		9.57	8.36	7.79	8.65	0.152	0.009
EDI-2: Drive for thinness		11.78	7.14	11.71	6.45	0.946	0.001
EDI-2: Body dissatisfaction		15.22	8.17	14.74	8.84	0.700	0.001
EDI-2: Interoceptive awareness		10.39	6.59	9.73	6.89	0.504	0.002
EDI-2: Bulimia		6.28	5.85	5.41	5.34	0.273	0.005
EDI-2: Interpersonal distrust		5.39	4.61	5.74	5.23	0.630	0.001
EDI-2: Ineffectiveness		10.84	7.08	9.40	7.24	0.169	0.008
EDI-2: Maturity fears		8.37	6.09	7.48	5.19	0.257	0.006
EDI-2: Perfectionism		6.07	4.89	5.05	4.08	0.103	0.011
EDI-2: Impulse regulation		5.43	5.30	5.36	5.81	0.925	0.001
EDI-2: Ascetic		6.70	4.24	6.05	4.25	0.289	0.005
EDI-2: Social insecurity		7.00	4.66	6.90	5.57	0.901	0.001
EDI-2: Total score		93.48	46.79	87.56	45.95	0.377	0.003
SCL-90R: Somatization		1.78	1.02	1.60	0.90	0.198	0.007
SCL-90R: Obsessive/compulsive		1.78	0.97	1.73	0.92	0.734	0.001
SCL-90R: Interpersonal sensitivity		1.90	0.99	1.88	1.00	0.848	0.001
SCL-90R: Depressive		2.25	0.98	2.06	0.99	0.196	0.007
SCL-90R: Anxiety		1.63	0.91	1.46	0.91	0.194	0.007
SCL-90R: Hostility		1.17	0.88	1.19	0.90	0.898	0.001
SCL-90R: Phobic anxiety		0.84	0.86	0.88	0.91	0.736	0.001
SCL-90R: Paranoid Ideation		1.43	0.89	1.28	0.84	0.253	0.006
SCL-90R: Psychotic		1.36	0.82	1.17	0.72	0.081	0.013
SCL-90R: GSI score		1.69	0.78	1.58	0.78	0.300	0.005
SCL-90R: PST score		61.43	19.63	60.36	18.86	0.697	0.001
SCL-90R: PSDI score		2.35	0.59	2.22	0.61	0.127	0.010

Note. EDI-2: V: Cramer’s-V. η^2^: partial eta-squared. Eating Disorders Inventory-2 [49]. SCL-90R: Symptom Checklist—Revised [48]. GSI: Global Severity Index. PST: Positive Symptom Total. PSDI: Positive Symptom Distress Index.

## Data Availability

Data are not available in any repository. Contact with the corresponding author.

## Supplementary Materials

The following are available online at https://www.mdpi.com/article/10.3390/nu13072145/s1, Table S1: Descriptives of the sample.
