# Supplementary material for: Neuropsychological Learning Deficits as Predictors of Treatment Outcome in Patients with Eating Disorders
Source: Nutrients. 2021 Jun 23;13(7):2145. doi: 10.3390/nu13072145 (PMC8308216; doi:10.3390/nu13072145)
Supplement: Supplementary file 1 [file nutrients-13-02145-s001.zip › nutrients-1211452-supplementary.pdf]

Table S1 (supplementary) Descriptives of the sample

		HC *(n=191)*		ED *(n=233)*		
		Mean	SD	Mean	SD	*p*
Chronological age (years-old)		25.65	8.50	30.52	10.90	**<.001***
		n	*%*	*n*	*%*	
Sex	Women	151	79.1%	190	81.5%	.521
	Men	40	20.9%	43	18.5%	
Marital status	Single	145	75.9%	164	70.4%	.144
	Married - partner	40	20.9%	52	22.3%	
	Divorced - separated	6	3.1%	17	7.3%	
Education	Primary	11	5.8%	82	35.2%	**<.001***
	Secondary	107	56.0%	95	40.8%	
	University	73	38.2%	56	24.0%	
Employment	Unemployed	52	27.2%	97	41.6%	**.002***
	Employed / Student	139	72.8%	136	58.4%	

Note. HC: healthy control. ED: eating disorder. SD: standard deviation.
